# Delayed mycotic aneurysm of the superficial temporal artery

**DOI:** 10.1016/j.jvscit.2026.102271

**Published:** 2026-04-21

**Authors:** Claire Heigl Maza, Jordan Kuhlman, Gabriela Benitez, Ryan Turley

**Affiliations:** Texas A&M University Naresh K. Vashisht College of Medicine, Round Rock, TX

**Keywords:** Aneurysm, Mycotic aneurysm, Infected aneurysm, External carotid artery

## Abstract

External carotid artery aneurysms are rare, and mycotic involvement is even more uncommon. We present a 65-year-old man who developed a rapidly enlarging mycotic superficial temporal artery aneurysm 2 years after blunt trauma. Initial imaging showed only a minor temporal bone fracture. During a 2023 hospitalization for small bowel obstruction complicated by ileus, intubation, and empiric antibiotics, magnetic resonance imaging revealed a 1.85-cm superficial temporal artery aneurysm, which enlarged to 2.69 cm during a later admission. He presented with rapid expansion and skin breakdown and underwent urgent open excision, followed by culture-directed oral antibiotics for 2 weeks, resulting in complete resolution.

External carotid artery aneurysms are rare and most commonly result from trauma, atherosclerosis, or vasculitis.^1^ Mycotic aneurysms of the superficial temporal artery (STA) represent an exceptionally uncommon entity, with STA pseudoaneurysms accounting for less than 1% of all vascular lesions. Infected extracranial aneurysms are themselves rare, and involvement of the STA is extraordinarily unusual. Most STA aneurysms arise from blunt trauma or iatrogenic injury, whereas secondary infection leading to mycotic transformation is exceedingly rare.^2^ Owing to the scarcity of these lesions, no standardized management guidelines exist with no established size criteria for elective repair, and there are no randomized studies comparing antimicrobial therapy alone with endovascular or surgical approaches. Current treatment strategies typically include antimicrobial therapy, endovascular intervention, or surgical excision, alone or in combination.^3^ Treatment decisions are based on clinical presentation, symptoms, and patient factors rather than specific size thresholds.^3^, ^4^, ^5^

We report a unique case of a mycotic STA pseudoaneurysm managed with open excision and culture-directed oral antibiotics, resulting in complete resolution and excellent clinical outcome.

## Case presentation

A 65-year-old man presented with a rapidly enlarging, painful right temporal mass. His history was notable for blunt trauma to the right temporal region in 2021 after being struck by a baseball traveling approximately 100 mph. Initial head computed tomography demonstrated a minor right squamosal temporal bone fracture without hemorrhage or vascular injury, and he was discharged with outpatient follow-up.

Nearly 2 years later, the patient was hospitalized for small bowel obstruction. His medical history included prior partial colectomy for diverticulitis. After failed conservative management, he underwent robotic-assisted adhesiolysis and small bowel resection. His postoperative course was complicated by prolonged ileus, transient intubation, fluctuating mental status, and small intraabdominal fluid collections. He received empiric broad-spectrum antibiotics; no cultures were obtained.

**Fig 1:**
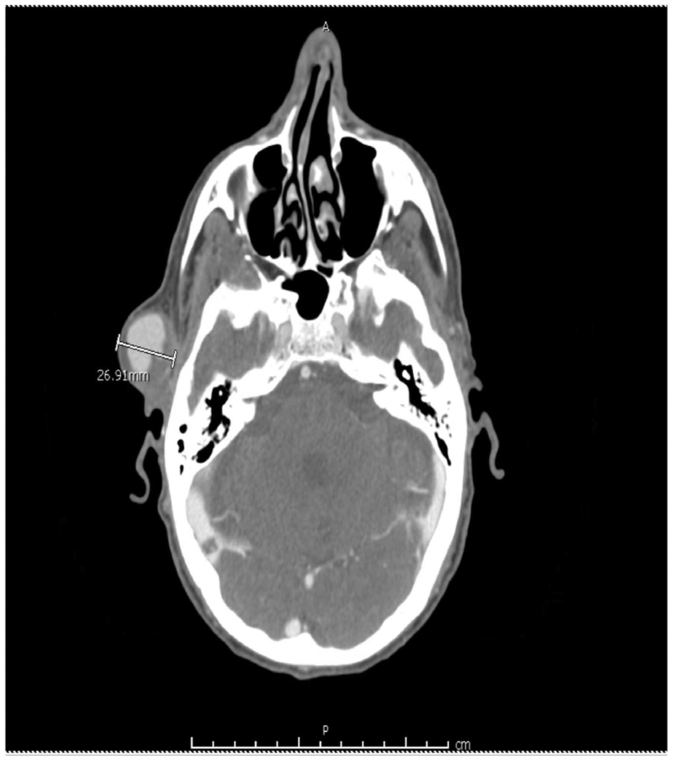
Computed tomography angiography demonstrates increased size of the right superficial temporal artery (STA) aneurysm to 2.69 cm.

During evaluation for altered mental status, brain magnetic resonance imaging incidentally revealed a 1.85-cm right STA aneurysm. The follow-up plan for the 1.85-cm STA aneurysm was unclear based on available outside hospital records, and it was presumed at that time that the aneurysm was not mycotic. Before outpatient follow-up, the patient re-presented to the same outside hospital with worsening COVID pneumonia. Repeat computed tomography angiography demonstrated interval enlargement of the aneurysm to 2.69 cm (Fig 1). Due to the lack of inpatient vascular surgery coverage, and in the absence of skin necrosis or pain during recovery from COVID pneumonia, the patient was referred for outpatient evaluation.

**Fig 2:**
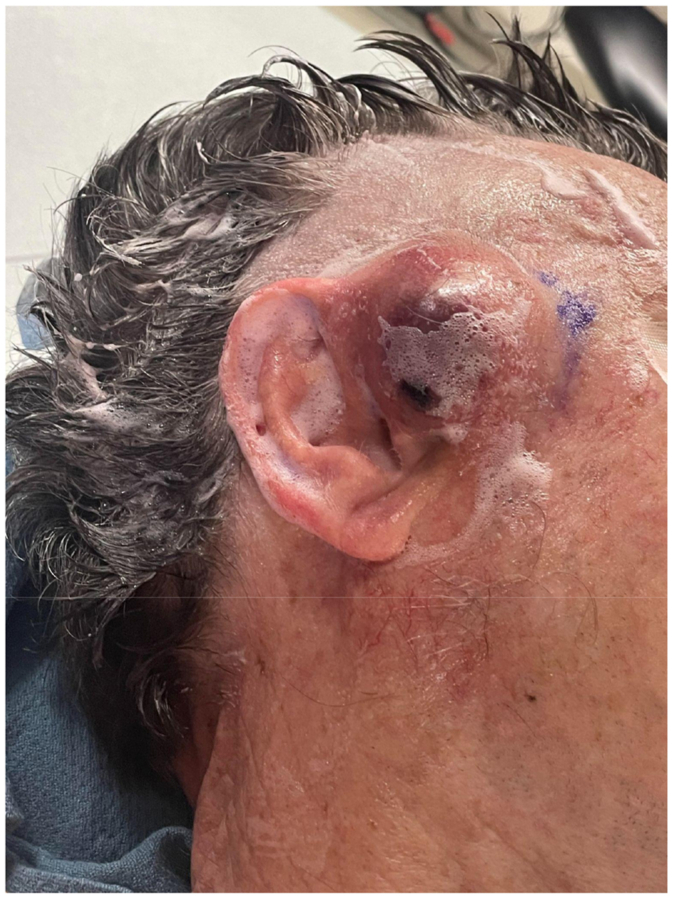
Preoperative images of the superficial temporal artery (STA) aneurysm.

**Fig 3:**
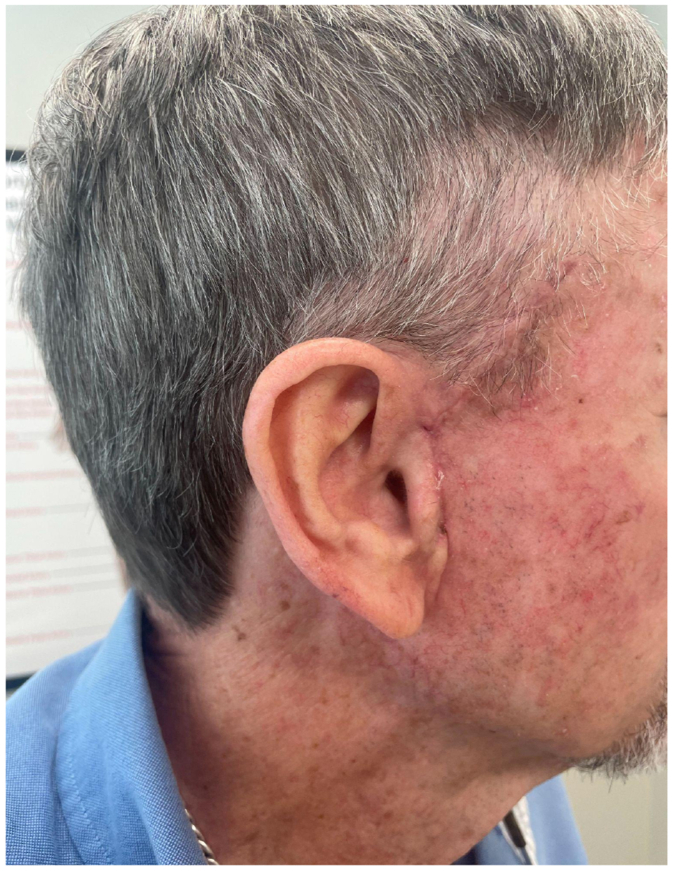
Postoperative image of the superficial temporal artery (STA) aneurysm repair.

He subsequently presented to our clinic for the first time with clinical evidence of further expansion, including associated tenderness and overlying skin breakdown (Fig 2). Given the rapid progression after his recent complex hospitalization, there was concern for an unrecognized mycotic extracranial arterial aneurysm. Urgent open excision was recommended, and the patient was immediately scheduled for surgery. A longitudinal incision medial to the ear was made, skin flaps were elevated, and arterial Doppler confirmed STA inflow and outflow. The aneurysm was excised in its entirety. Because of thinning of the overlying skin, limited local tissue rearrangement was required for closure. Wound cultures grew *Staphylococcus epidermidis*, sensitive to sulfamethoxazole-trimethoprim. The patient completed a 2-week course of oral antibiotics. At follow-up, he demonstrated complete wound healing without evidence of recurrence or ongoing infection (Fig 3). Surveillance examination at subsequent clinic visits showed no residual mass or vascular abnormality.

## Discussion

STA pseudoaneurysms typically present as painless, pulsatile temporal masses within weeks to months after blunt head trauma.^6^^,^^7^ The differential diagnosis includes hematoma, abscess, lipoma, sebaceous cyst, lymphadenopathy, and soft tissue tumors, emphasizing the importance of vascular imaging in patients with enlarging temporal lesions. Duplex ultrasound examination may demonstrate characteristic “yin-yang” flow, whereas computed tomography angiography provides detailed anatomic assessment and operative planning.

This case represents an unusually delayed presentation of traumatic STA pseudoaneurysm complicated by secondary infection. In contrast to typical presentations, this patient exhibited nearly a 2-year latency between injury and diagnosis, which is highly atypical.

Traumatic STA pseudoaneurysms result from disruption of the arterial wall, leading to blood extravasation and formation of a fibrous capsule lacking normal vessel layers.^7^^,^^8^ Unlike true aneurysms, pseudoaneurysms do not contain all three arterial wall layers and are almost exclusively traumatic.^5^ The temporal bone fracture documented in 2021 likely initiated arterial injury, with gradual expansion accounting for delayed clinical manifestation.

Rapid interval growth occurred over approximately 8 weeks, coinciding with severe systemic illness. Although growth dynamics of STA pseudoaneurysms are not well characterized, systemic inflammatory states may contribute to vascular instability. COVID-19 infection is associated with endothelial dysfunction and inflammatory vasculopathy, which may plausibly accelerate aneurysmal expansion.^9^, ^10^, ^11^, ^12^ In addition, the patient's complicated postoperative course after bowel surgery may have further amplified systemic inflammation and impaired tissue healing.

Isolation of *S epidermidis* represents an uncommon finding in this setting. Although frequently regarded as a skin commensal, *S epidermidis* is a recognized cause of vascular infection, particularly in compromised tissues.^13^, ^14^, ^15^, ^16^ The clinical presentation of rapid enlargement, tenderness, and skin breakdown suggests true infection rather than contamination. The patient's recent hospitalization and exposure to broad-spectrum antibiotics may also have altered his skin microbiome, facilitating opportunistic infection. Successful treatment with culture-directed oral antibiotics supports the clinical relevance of this finding.

Urgent surgical excision was indicated given rapid growth, pain, skin compromise, and concern for rupture. Based on our review of the current body of literature, no specific guidelines regarding size criteria for resection could be identified. Although STA pseudoaneurysms rarely result in life-threatening hemorrhage, rupture can cause significant morbidity, cosmetic deformity, and difficult-to-control bleeding.^4^^,^^5^ Surgical ligation and excision remain the preferred treatment, offering definitive management and allowing tissue sampling for microbiologic analysis. A systematic review reported successful surgical resection in 128 cases with only one recurrence. Endovascular approaches, including coil embolization or liquid embolic agents, may be considered in select patients with prohibitive operative risk; however, open excision provides durable results and diagnostic confirmation.^4^^,^^5^

This case underscores several important clinical considerations. Traumatic STA pseudoaneurysms may present after prolonged latency, warranting continued vigilance in patients with prior craniofacial trauma. Secondary infection, although rare, should be suspected in cases of rapid enlargement, pain, or skin changes. Finally, close surveillance of incidentally discovered vascular lesions is critical, as earlier intervention may prevent complications necessitating urgent surgery.

## Conclusions

In conclusion, we successfully treated a mycotic aneurysm caused by *S epidermidis* with open excision, followed by a 2-week course of culture-directed oral antibiotic therapy.

## Patient consent

Informed consent was obtained from the patient for the publication of this case report and any accompanying images.

## Funding

None.

## Disclosures

None.
